# A Retrospective Longitudinal Within-Subject Risk Interval Analysis of Immunoglobulin Treatment for Recurrent Acute Exacerbation of Chronic Obstructive Pulmonary Disease

**DOI:** 10.1371/journal.pone.0142205

**Published:** 2015-11-11

**Authors:** Juthaporn Cowan, Logan Gaudet, Sunita Mulpuru, Vicente Corrales-Medina, Steven Hawken, Chris Cameron, Shawn D. Aaron, D. William Cameron

**Affiliations:** 1 Division of Infectious Diseases, Department of Medicine, University of Ottawa, Ottawa, Ontario, Canada; 2 Division of Respirology, Department of Medicine, University of Ottawa, Ottawa, Ontario, Canada; 3 Clinical Epidemiology Program, The Ottawa Hospital Research Institute, Ottawa, Ontario, Canada; Zhongshan Hospital Fudan University, CHINA

## Abstract

**Background:**

Recurrent acute exacerbations of chronic obstructive pulmonary disease (AECOPD) are common, debilitating, costly and often difficult to prevent.

**Methods:**

We reviewed records of patients who had COPD and immunoglobulin (Ig) treatment as adjunctive preventative treatment for AECOPD, and documented all AECOPD episodes for one year before and after initiation of Ig treatment. We graded AECOPD episodes as moderate for prescription of antibiotics and/or corticosteroids or for visit to the Emergency Department, and as severe for hospital admission. We conducted a retrospective within-subject self-controlled risk interval analysis to compare the outcome of annual AECOPD rate before and after treatment.

**Results:**

We identified 22 cases of certain COPD, of which three had early discontinuation of Ig treatment due to rash and local swelling to subcutaneous Ig, and five had incomplete records leaving 14 cases for analyses. The median baseline IgG level was 5.9 g/L (interquartile range 4.1–7.4). Eight had CT radiographic bronchiectasis. Overall, the incidence of AECOPD was consistently and significantly reduced in frequency from mean 4.7 (± 3.1) per patient-year before, to 0.6 (± 1.0) after the Ig treatment (*p* = 0.0001). There were twelve episodes of severe AECOPD (in seven cases) in the year prior, and one in the year after Ig treatment initiation (*p* = 0.016).

**Conclusions:**

Ig treatment appears to decrease the frequency of moderate and severe recurrent AECOPD. A prospective, controlled evaluation of adjunctive Ig treatment to standard therapy of recurrent AECOPD is warranted.

## Introduction

Patients with COPD are prone to recurrent episodes of acute exacerbation (AECOPD), which have short- and long-term morbidity and mortality [1–4]. Patients with frequent AECOPD use healthcare services disproportionately, with increased healthcare costs [5,6]. The majority of this cost is driven by the exacerbations for which patients are hospitalized [7]. Therefore, interventions to reduce the frequency of exacerbation and subsequent healthcare use would have a significant impact on healthcare costs, as well as health and quality of life. However, since the presence and severity of comorbidities increases mortality risk [8], reduction of AECOPD might not directly improve mortality.

Stable COPD is characterized by the presence of increased numbers of inflammatory cells in airways [9]. AECOPD is a result of inflammatory process triggered mostly by respiratory viral and/or bacterial airway infection [10]. Several non-steroidal immunomodulatory agents have been tried as adjuvant therapy (in addition to inhaled bronchodilators and corticosteroids) in attempts to further reduce the frequency of AECOPD but failed to elicit significant efficacy [11–15]. Macrolides have anti-inflammatory effect [16] and are used in patients with severe COPD and a history of frequent exacerbations [17]. However, long-term macrolide therapy is associated with risk of microbial resistance and cardiovascular adverse effects [18–20]. The development of newer immunomodulatory agents as adjuvant therapy to prevent AECOPD has become an area of intense investigation [21,22].

Prolonged steroid use is associated with hypogammaglobulinemia in asthmatic patients [23]. Patients with COPD have lower immunoglobulin G (IgG) level than patients with other lung diseases independent of oral steroid use and age [24]. Intravenous and subcutaneous immunoglobulins (IVIg and SCIg, respectively) are prepared from pooled plasma from thousands of healthy blood donors. The large donor pool ensures a diversity of antibody specificities to a wide spectrum of antigens and microbial pathogens [25]. IVIg or SCIg represents a privileged source of natural antibodies (NAb), occur in the absence of autoimmune disease or immunization. NAb are not only an immune defense against pathogens [26] but also have anti-inflammatory and immunomodulatory activities [27,28]. Given the heightened systemic and airway inflammatory activity in patients with COPD, their propensity to infection-triggered AECOPD, and their suppressed mucosal or systemic immunity [29,30], the anti-inflammatory, anti-infective and immunomodulatory effects of Ig preparations could be beneficial in this group. A clinical trial of IVIg as adjunctive treatment in hospitalized COPD patients with sputum cultures positive for fungi appeared to reduce average length of hospital stay and mortality at six months [31]. However, to our knowledge, there has not been a study looking at the effect of Ig treatment in the prevention of AECOPD.

In our institution, we established a clinical program for Ig treatment, and we identified patients who had been placed on Ig treatment as an adjunctive preventative measure for recurrent AECOPD. Most patients reported subjective improvement with less frequent exacerbation and were disinclined to discontinue treatment despite lack of clear indication. We conducted a retrospective longitudinal observational within-subject risk-period analysis of all COPD patients treated with immunoglobulin, to objectively measure the number and severity of AECOPD, and to compare rates in the years before and after initiation of Ig treatment.

## Methods

### Study design

This is a retrospective longitudinal case-only self-controlled risk-interval analysis to compare the number of AECOPD the year before and after initiation of Ig treatment for patients with COPD. This analytical method removes the potential confounding effect of fixed covariates, although is susceptible to other biases, such as temporal confounding [32]. This study received research ethics board approval from the Ottawa Hospital Research Ethics Board (OHREB) prior to data abstraction (OHREB# 20140346-01H). The OHREB waived the need for written patient consent. Any patient identifiers were not used and all data were anonymized.

### Study population and setting

All adult cases who had Ig treatment and had a clinical diagnosis of COPD at The Ottawa Hospital, a tertiary care teaching hospital of the University of Ottawa, between 2008 and 2014 were identified from medical and transfusion records. According to the GOLD criteria (www.goldcopd.org), we then excluded cases who did not have medical record documentation of COPD based on diagnostic lung function indices (FEV1 < 80 percent of predicted value, and FEV1/FVC < 70) before the initiation of Ig treatment. We assessed all confirmed cases for treatment tolerance. However, for comparisons of AECOPD rates before and after initiation of Ig treatment, we excluded cases with inadequate documentation of AECOPD before Ig treatment, and those who received Ig treatment for less than three months.

### Study outcomes

Our primary outcome was the one-year incidence rate and severity of AECOPD before and after initiation of Ig treatment. We graded AECOPD episodes as moderate if they triggered the use of prescribed oral antibiotics and/or corticosteroids, or a visit to the Emergency Department (ED), and as severe if they triggered hospital admission.

We also collected information regarding route of Ig treatment, dose, tolerance and adverse effects.

### Data analysis

We used a retrospective longitudinal case-only self-controlled risk-interval analysis to compare the rates of moderate and severe AECOPD in the one-year period before and after the initiation of Ig treatment. Variables were described using means/medians and standard deviations (SD)/range. Comparisons of within-patient AECOPD rates were made using non-parametric paired difference tests (Sign test and Wilcoxon signed rank tests). All tests were 2-sided and a p value of less than 0.05 was considered statistically significant. Analyses were implemented using SAS-v.9.3 and R statistical software.

## Results

### Study population and treatment

**Fig 1:**
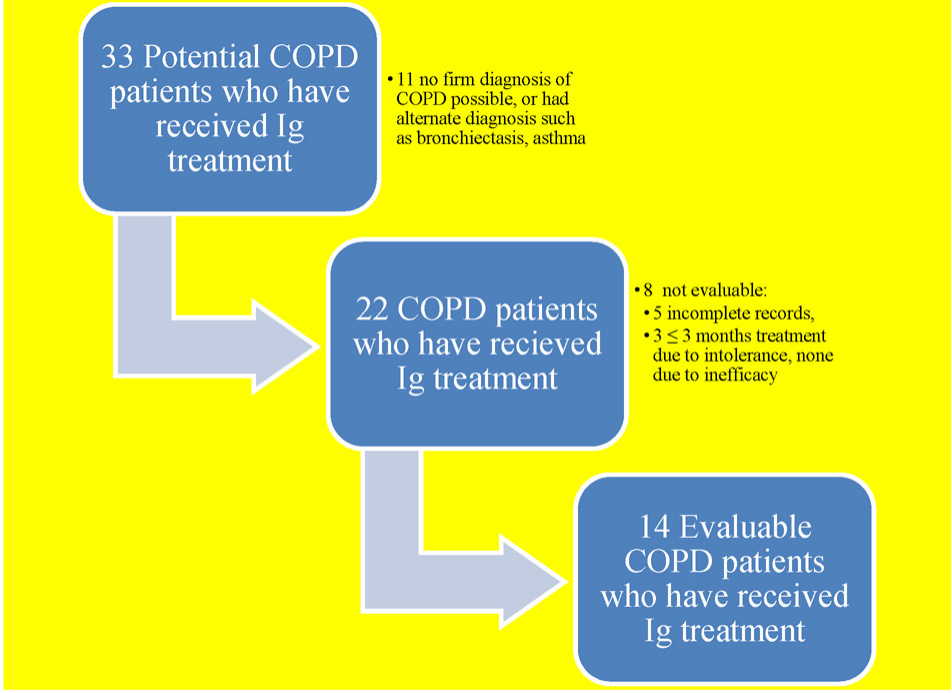
Flowchart of study population identified for analysis.

We identified 33 cases started on Ig treatment for prevention of frequent recurrent AECOPD (Fig 1) at our institution. Of these 33 cases, eleven did not meet spirometric criteria of COPD diagnosis, and were excluded. Of the remaining 22 cases, five had incomplete records of AECOPD in the pre-treatment period, and three had early discontinuation of treatment due to adverse reactions to Ig treatment. There were no instances of Ig treatment discontinuation due to inefficacy. Ultimately, there were 14 evaluable cases for our primary analysis (Fig 1).

Demographic, clinical and laboratory characteristics of the fourteen evaluable cases included in our primary analysis are detailed in Table 1. The mean (± SD) age was 67 (± 12) years. There were six male and eight female cases. COPD was the clinically dominant morbidity in every case. The mean FEV1 was 46.3% (± 18.6%) of predicted value, and FEV1/FVC was 43.4 (± 15.3). Half (8) had at least severe COPD (S1 Appendix) by GOLD criteria. Bronchiectasis on computed tomography scan was present in eight cases, and did not correlate with severity of COPD. Asthma was present in seven cases. Approximately one third (5) of cases were obese (BMI > 30 kg/m^2^) and three were morbidly obese (BMI > 35 kg/m^2^). Two had coronary artery disease, one had diabetes mellitus, and one had monoclonal gammopathy. Mean serum IgG level was 6.2 (± 2.2), median 5.9 g/L; nine (64.3%) had hypogammaglobulinemia (serum IgG < 7 g/L) documented before Ig treatment, but only five had potentially significant hypogammaglobulinemia (IgG < 5 g/L). The mean Ig treatment dose was 0.5 ± 0.3 g/kg/month. Six (42.9%) cases received IVIg, seven (50%) received SCIg, and one case was switched from IVIg to SCIg due to intolerance to the former. Of fourteen cases under observation, one completed only eleven treatment months, interrupted for unrelated personal stress. The overall mean duration of observed treatment in fourteen cases was 363.1 (± 6.9) days.

**Table 1:** Demographic data.

	Means ± Standard Deviation
Age (years)	67.1±12.1
Sex (M:F)	43:57
Body mass index	30.4±9.4
FEV1 (L/sec)	1.2±0.8
FEV1%	46.3±18.6
FEV1/FVC	43.4±15.3
Total IgG (g/L)	6.2±2.2
Ig administration	IV:SC 6:7*
Dosage (g/kg)	0.5±0.3

IV = intravenous

SC = subcutaneous

FEV1 –forced expiratory volume in 1 second

FEV1%—percentage of average normal predicted FEV1 value

FVC–forced vital capacity

*one person switched from IV to SC

### Rate of AECOPD events before and after initiation of immunoglobulin treatment

**Fig 2:**
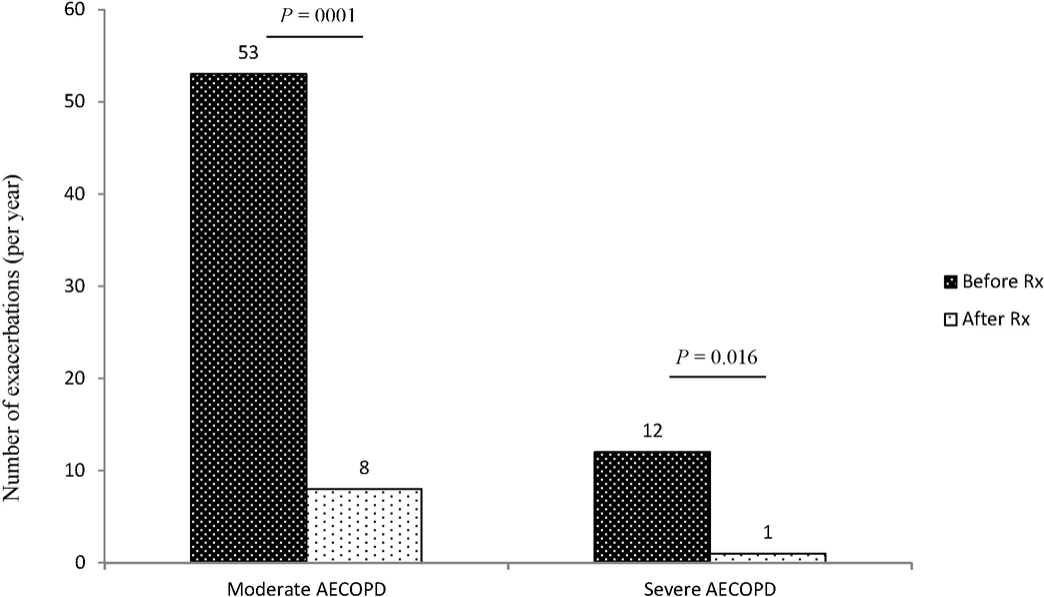
Total number of exacerbations per year in each class of exacerbation the year before and the year after Ig treatment. Black bars represent numbers of exacerbations before Ig treatment. White bars represent numbers of exacerbations after Ig treatment. See Methods section for definition of each class of COPD exacerbation. The overall decrease in number of exacerbations ignoring the clustering of events among patients was an 84.9% reduction in moderate AECOPD events, 91.7% in severe AECOPD events and 86.2% in moderate or severe AECOPD.

The mean rates of moderate or severe AECOPD events before and after initiation of Ig treatment were 4.65 and 0.64 per patient-year observation, respectively (p = 0.0001, Table 2). Mean and median reduction of moderate or severe AECOPD events by patient was 81.1% and 100%, respectively (Table 2). The lowest percent reduction by patient in our cohort was 25%. Overall, there was 86.2% reduction in the annualized rate (65 VS 9 actual AECOPD events, Fig 2).

**Table 2:** COPD exacerbations before and after Ig treatment. Average exacerbations per patient-year and observed reduction in each class of exacerbation*.

	Before		After		Mean/Median (range) of % decrease in events by patient	P-value (Wilcoxon signed rank test) before vs. after
	Total events (n = 14 patients)	Mean/Median (range) of events/patient-year	Total events (n = 14 patients)	Mean/Median (range) of events/patient-year		
Moderate AECOPD	53	3.8/3.0 (0–12)	8	0.57/0 (0–3)	66.7%/91.7% (0%– 100%)	0.001
Severe AECOPD	12	0.86/0.5 (0–2)	1	0.07/0 (0–1)	46.4%/25.0% (0%– 100)	0.016
**Moderate or severe AECOPD**	**65**	**4.65/ 4.0 (2–12)**	**9**	**0.64/0 (0–3)**	**81.1%/100% (25.0%– 100%)**	**0.0001**

* %decrease = (events after–events before)/events before. No patients experienced an increase in events.

The reported ranges report the highest and lowest events/patient year by patient, and the highest and lowest % decrease observed by patient.

Mean and median % decreases were calculated by patient, therefore, this would not correspond to overall % decrease in events.

**Fig 3:**
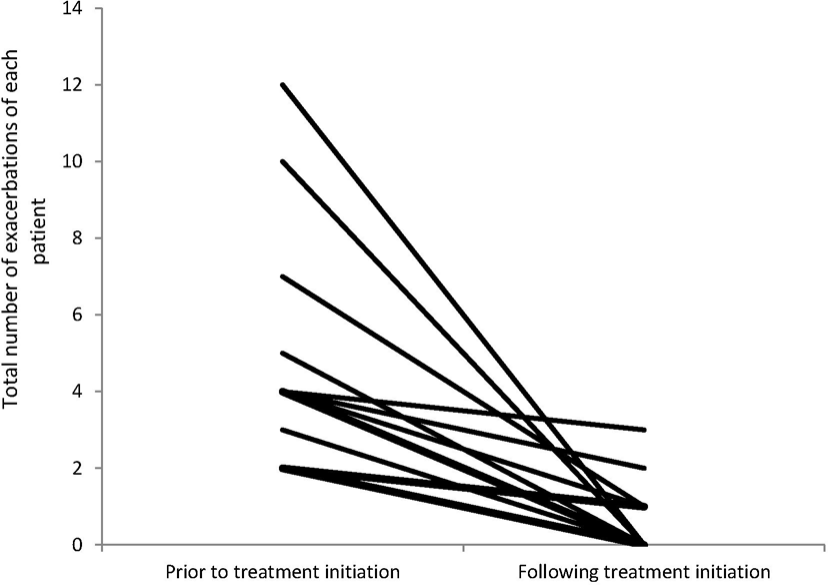
All exacerbations per year before and after Ig treatment in each studied patient.

The rate of moderate AECOPD decreased from 3.8 to 0.57 per patient-year (53 VS 8 episodes, p = 0.001, Table 2 and Fig 2). The rate of hospitalization for severe AECOPD decreased from 0.86 to 0.07 (12 VS 1 hospital admissions, p = 0.016 Table 2 and Fig 2). Twelve hospital admissions occurred in seven of fourteen cases before Ig treatment, and a single hospitalization on treatment. Fig 3 illustrates consistent reduction in the annual rate of all AECOPD in each individual case after Ig treatment. The reduction in exacerbations remained statistically significant after 1) excluding the one patient who was followed for slightly less than a full year on treatment, and 2) excluding two patients who experienced extremely high (10 or more) exacerbations. Very similar levels of statistical significance were observed for paired difference tests utilizing the Wilcoxon signed rank test and the more conservative and robust sign test.

A similar reduction in mild AECOPD also observed across all cases but not reported because of subjective nature of the data. The overall rate of AECOPD decreased consistently, across of severity of COPD, bronchiectasis or baseline serum level of IgG (Table 3).

**Table 3:** Rate of AECOPD events before and after Ig treatment classified by stage of COPD based on GOLD criteria, presence or absence of radiographic bronchiectasis, or baseline IgG level.

	Before (AECOPD events/patient-year)	After (AECOPD events/patient-year)
***Stage of COPD***		
Moderate COPD (n = 7)	5.14 ± 2.85	0.86 ± 1.13
Severe / very severe COPD	4.14 ± 2.95	0.43 ± 0.5
***Presence of bronchiectasis***		
No bronchiectasis (n = 6)	4.00 ± 2.83	0.83 ± 1.07
With bronchiectasis	5.13 ± 2.93	0.50 ± 0.71
***Baseline IgG level***		
IgG ≥ 5.9 g/L (n = 7)	4.29 ± 3.33	0.29 ± 0.45
IgG < 5.9 g/L	5.00 ± 2.45	1.00 ± 1.07

IgG = 5.9 g/L is the median IgG in our case series.

### Adverse events

Of the 22 cases with spirometric evidence of COPD initially considered for analysis of AECOPD incidence, three experienced adverse reactions to the first dose of SCIg injection and were excluded due to brevity of treatment (Fig 1). Two of these had local swelling and respiratory symptoms with hoarseness or shortness of breath. Neither of these patients presented at the ED, but improved on inhaled corticosteroids and anti-histamine. The adverse reaction in the remaining case was only local swelling. This patient was re-challenged after six months and tolerated treatment well thereafter. Among the fourteen cases included in our primary analysis, one had transfusion reaction to IVIg that manifested as fever and rigors, which abated with intravenous corticosteroid. The patient subsequently responded favorably to SCIg without any local or systemic reaction. The remaining thirteen cases did not report any adverse reaction.

## Discussion

This study has important strengths and limitations. Strengths of our study are that the non-parametric within-subject analysis controlled for all fixed baseline covariates and was robust to departure from normality and free of other distributional assumptions about the population from which our patients originated. The effect size we observed was large in magnitude and highly clinically and statistically significant using a robust paired difference tests, the sign test and Wilcoxon signed rank test.

Limitations of our study are that it is not a prospective randomized controlled trial, that it only includes the experience of few prescribers in a single center, and a small but inclusive series of cases. The hospitalizations, ED visits and the use of antibiotics and/or steroids were both self-reported and clinically recorded or documented by health care providers, but recall and documentation biases remain a possibility. It is possible that we did not capture ED visits and hospitalization in other hospitals, although these were sought. However, selection bias for analysis of efficacy was limited, as all Ig treatment cases in this regional tertiary care centre were identified by means of review of centralized transfusion documentation. Furthermore, eight (of 22) COPD cases excluded from analysis did not discontinue treatment due to inefficacy, but rather due to intolerance and short observation, or absent documentation prior to treatment.

Our cases were reviewed for syndromic diagnosis (by SDA and DWC) at inclusion, and the dominant clinical syndrome in each was COPD, not bronchiectasis or immunodeficiency. A third of our study population was obese. Half had radiographic bronchiectasis, consistent with the reported prevalence of bronchiectasis in patients with COPD [33]. Five (36%) had IgG levels below 5 gm/L (one with monoclonal gammopathy), and these patients were not screened for vaccine hyporesponsiveness prior to Ig treatment to distinguish primary or secondary immunodeficiency from COPD- and treatment-associated hypogammaglobulinemia [23,24]. These may be representative of the known heterogeneity of COPD and co-morbidities in general [8]. Our sample is too small to explore covariates of treatment response; however, the response seems to be consistent across cases and co-morbidity in our study.

Inter-current treatment is an unlikely confounder, as our patients were on stable therapies and the effect size observed is greater than the effectiveness of proven therapies. One might argue that the reduction in observed rate is a result of regression to the mean, or due to seasonality or annual differences in exacerbation risk. For the latter, we included two full calendar years of observation of each case, and this occurred over the span of about six years, which reduces the likelihood of temporal variation accounting for such a consistent observation (S3 Appendix). For the former, as the severity of COPD increases, exacerbations and need for hospitalization become more frequent over time [34]. In a large prospective three-year cohort study, the basal rate of exacerbation in patients with severe and very severe COPD over one year was 1.34 per person, and 2.0 per person, respectively [34]. This study also demonstrated that having a prior exacerbation increased the odds of having an exacerbation in the following year by 4.3 times [34]. We also point out that the baseline rate of exacerbation in patients with severe and very severe COPD in general is more than 1 per person-year, which is higher than what we observed in our case series after Ig treatment [17,34]. With this expected basal constancy and incremental risk of recurrence, it is unlikely that our result is regression to the mean.

In summary of strengths and limitations, the consistent and clinically important apparent effect size observed here does not need as much in statistical analysis to convince one against chance, as it needs external validation that representativeness, observation, detection or other biases might account for the finding.

In this study, Ig treatment was associated with a significant and consistent reduction in the frequency of AECOPD in a relatively small number of patients prone to frequent recurrence. This reduction was observed for both severe and moderate AECOPD episodes. There was no serious adverse event associated with Ig treatment in patients. We found that the annual rate of hospitalization for severe AECOPD decreased from 0.86 to 0.07. The average cost of a hospital admission in Ontario for AECOPD is approximately $6,000[35,36], while the costs for immunoglobulin (Ig) treatment using doses applied in this study range from approximately $15,000-$20,000 per year, depending on the price and dose of treatment (Communication with Canadian Blood Services). Accordingly, the cost associated with reduced hospitalizations may not offset the cost of Ig treatment, although this does not include the impact that Ig treatment has on health related quality of life, nor other costs. If proven effective, further research is needed on the cost-effectiveness of Ig treatment from the health care providers, payer and societal perspective.

Ig replacement therapy (monthly IVIg or weekly SCIg) is approved for use in patients with primary and secondary humoral immunodeficiency to prevent recurrent infection. Ig treatment is also used in other diseases such as multifocal motor neuropathy, Guillain-Barré syndrome, and other autoimmune diseases [28]. It is unclear to what degree Ig treatment effect in immunodeficiency is due to replenishing Nab and conferring anti-infective activity, or modulating inflammation and immune responses in autoimmunity. There have been few studies addressing these issues, for example, T-cell activation state in common variable immunodeficiency (CVID) patients can be alleviated by IVIg [37]. Physiologic dose of immunoglobulin accelerates phagocytosis, differentiation and maturation of dendritic cells *in vitro* [38]. IVIg can also temporarily reduce pro-inflammatory monocytes after infusion [39]. It is unknown; however, whether these effects are the same across very heterogeneous immunodeficiency cases. In the experimental mouse models, high dose immunoglobulin use in the form of IVIg has anti-inflammatory effect through Dendritic Cell-Specific Intercellular adhesion molecule-3-Grabbing Non-integrin (DC-SIGN) and promotion of Th2 response [40,41]. However, this finding is not seen in experiments using human dendritic cells [42]. In summary, the precise mechanisms through which anti-inflammatory and immunomodulatory activity takes place have not yet been completely elucidated [43].

Approximately fifty percent of AECOPD is triggered by virus [44]. Respiratory viral infection is also associated with bacterial superinfection [45]. Both viral and bacterial infections activate inflammatory cascades. The average pre-treatment IgG level in our primary cohort was not clinically deficient but it was raised from about six to ten g/L with treatment (data not shown). Reduction in AECOPD episodes was consistent in all cases regardless of Ig levels., Thus, the apparent effect of Ig treatment in our cases is not just due to replacement of Ig in the presence of deficiency. We hypothesize that immunoglobulin treatment may reduce COPD exacerbations by preventing viral and bacterial infection through the promotion of mucosal immunity [46], inhibiting the development of disease by natural antibodies [47], or by inhibiting a downstream inflammatory cascade triggered by these infections [48].

In summary, Ig treatment seems to reduce frequency of recurrent AECOPD to a clinically significant degree in this cases series. The internal consistency and magnitude of this association warrants external validation by replication and confirmation in prospective controlled evaluations.

## Supporting Information

S1 Appendix Baseline characteristics and Ig dosage. FEV1 = forced expiratory volume in 1 second. FEV1% = percentage of average normal predicted FEV1 value. FVC = forced vital capacity. BMI = body mass index.
(TIFF)

S2 Appendix Number of exacerbations before and after Ig treatment in each studied case. Follow up duration of each studied case is also included.
(TIFF)

S3 Appendix Graph of Influenza period in Eastern Ontario Region, Canada from year 2004/2005 to 2013/2014. (TIFF)

S4 Appendix STROBE Statement. Checklist of items that should be included in reports of observational studies.
(PDF)
