# Supplementary figures and images for: A Retrospective Longitudinal Within-Subject Risk Interval Analysis of Immunoglobulin Treatment for Recurrent Acute Exacerbation of Chronic Obstructive Pulmonary Disease
Source: PLoS One. 2015 Nov 11;10(11):e0142205. doi: 10.1371/journal.pone.0142205 (PMC4641695; doi:10.1371/journal.pone.0142205)

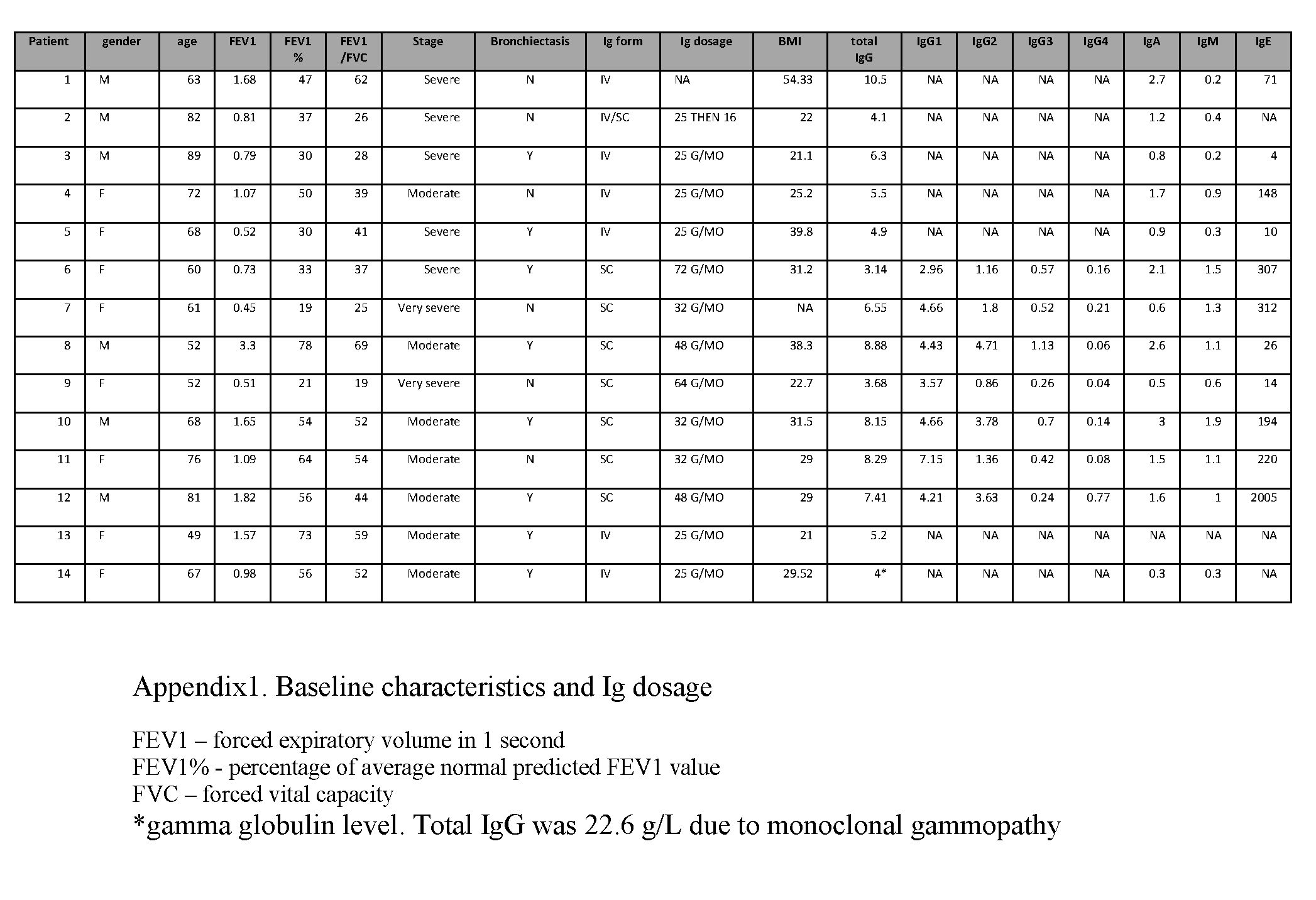

Supplement: S1 Appendix — FEV1 = forced expiratory volume in 1 second. FEV1% = percentage of average normal predicted FEV1 value. FVC = forced vital capacity. BMI = body mass index. (TIFF) [file pone.0142205.s001.tiff]

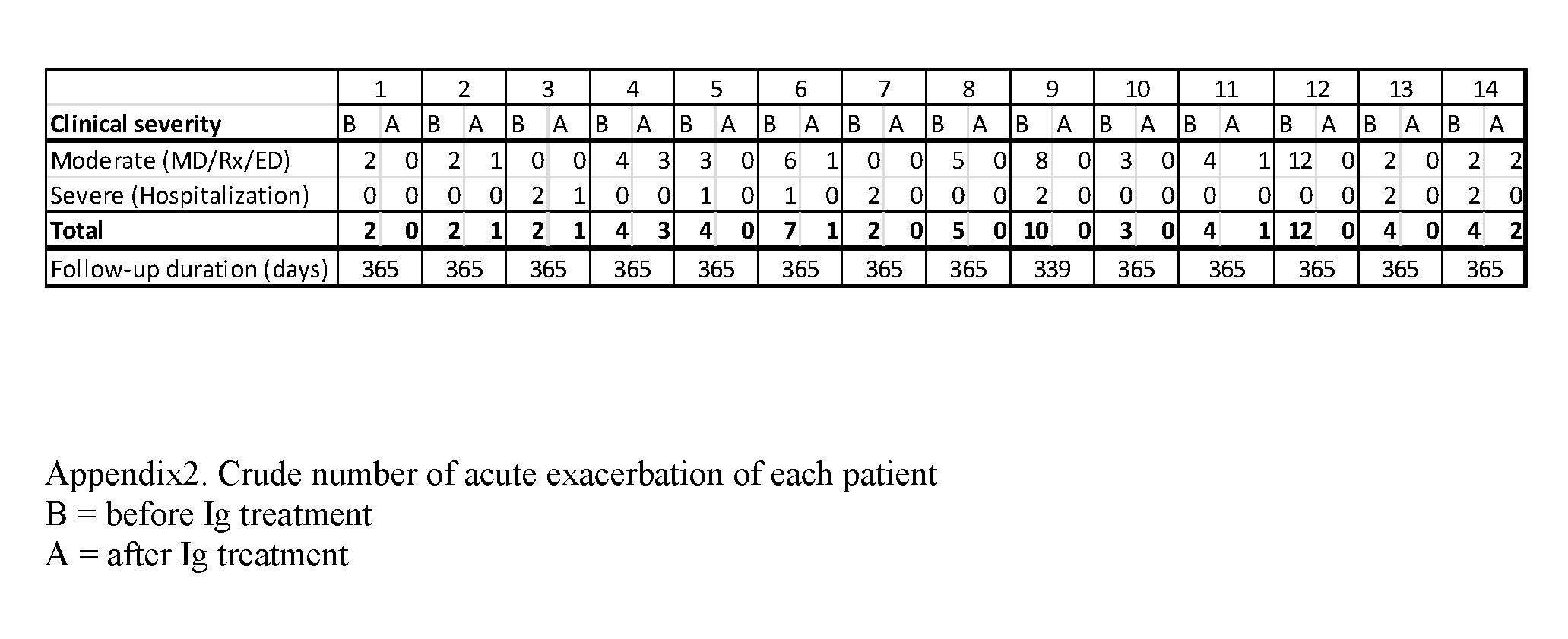

Supplement: S2 Appendix — Follow up duration of each studied case is also included. (TIFF) [file pone.0142205.s002.tiff]

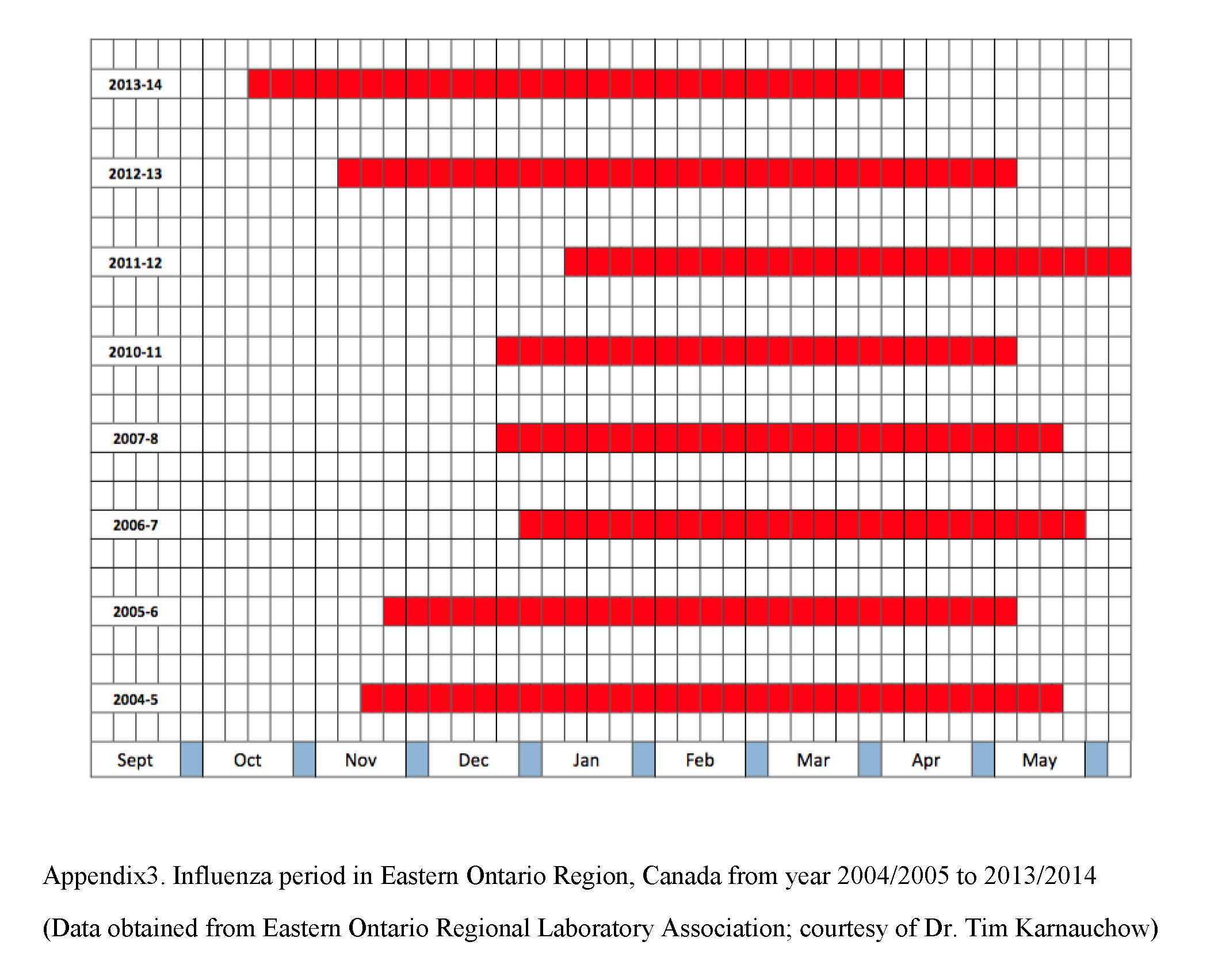

Supplement: S3 Appendix — (TIFF) [file pone.0142205.s003.tiff]
