# Supplementary material for: A Retrospective Longitudinal Within-Subject Risk Interval Analysis of Immunoglobulin Treatment for Recurrent Acute Exacerbation of Chronic Obstructive Pulmonary Disease
Source: PLoS One. 2015 Nov 11;10(11):e0142205. doi: 10.1371/journal.pone.0142205 (PMC4641695; doi:10.1371/journal.pone.0142205)
Supplement: S4 Appendix — Checklist of items that should be included in reports of observational studies. (PDF) [file pone.0142205.s004.pdf]

Appendix 4. STROBE Statement—checklist of items that should be included in reports of observational studies

	Item No.	Recommendation	Page No.	Relevant text from manuscript
**Title and abstract**	1	(*a*) Indicate the study's design with a commonly used term in the title or the abstract	1	Retrospective longitudinal within-subject analysis
		(*b*) Provide in the abstract an informative and balanced summary of what was done and what was found	3	Results and conclusions
**Introduction**				
Background/rationale	2	Explain the scientific background and rationale for the investigation being reported	4	Introduction
Objectives	3	State specific objectives, including any prespecified hypotheses	4	To objectively measure the number of exacerbations
**Methods**				
Study design	4	Present key elements of study design early in the paper	5	Self-controlled risk interval analysis
Setting	5	Describe the setting, locations, and relevant dates, including periods of recruitment, exposure, follow-up, and data collection	5	Study population and setting
Participants	6	(*a*) *Cohort study*—Give the eligibility criteria, and the sources and methods of selection of participants. Describe methods of follow-up	5	Study population and setting
		(*b*) *Cohort study*—For matched studies, give matching criteria and number of exposed and unexposed	N/A	
Variables	7	Clearly define all outcomes, exposures, predictors, potential confounders, and effect modifiers. Give diagnostic criteria, if applicable	5	Study outcomes
Data sources/ measurement	8*	For each variable of interest, give sources of data and details of methods of assessment (measurement). Describe comparability of assessment methods if there is more than one group	5	Chart review
Bias	9	Describe any efforts to address potential sources of bias	-	
Study size	10	Explain how the study size was arrived at	5	Study population and setting

	Item No.	Recommendation	Page No.	Relevant text from manuscript
Quantitative variables	11	Explain how quantitative variables were handled in the analyses. If applicable, describe which groupings were chosen and why	5	Data analysis
Statistical methods	12	(*a*) Describe all statistical methods, including those used to control for confounding	5	Data analysis
		(*b*) Describe any methods used to examine subgroups and interactions	N/A	
		(*c*) Explain how missing data were addressed	5	Study population and setting
		(*d*) *Cohort study*—If applicable, explain how loss to follow-up was addressed	N/A (no loss to follow-up)	
		(*e*) Describe any sensitivity analyses	N/A	
**Results**				
Participants	13*	(a) Report numbers of individuals at each stage of study—eg numbers potentially eligible, examined for eligibility, confirmed eligible, included in the study, completing follow-up, and analysed	5-6	Study population
		(b) Give reasons for non-participation at each stage	5-6	Study population
		(c) Consider use of a flow diagram	14	Flow chart
Descriptive data	14*	(a) Give characteristics of study participants (eg demographic, clinical, social) and information on exposures and potential confounders	5, 6, 15, 20	Demographic data, table1, appendix1
		(b) Indicate number of participants with missing data for each variable of interest	14	Eight inevaluable cases
		(c) *Cohort study*—Summarise follow-up time (eg, average and total amount)	6	Mean treatment-day
Outcome data	15*	*Cohort study*—Report numbers of outcome events or summary measures over time	6-7, 16-19	Rate of AECOPD, table 2, figure2&3
Main results	16	(*a*) Give unadjusted estimates and, if applicable, confounder-adjusted estimates and their precision (eg, 95% confidence interval). Make clear which confounders were adjusted for and why they were included	6	Total number of exacerbations per year
		(*b*) Report category boundaries when continuous variables were categorized	N/A	
		(*c*) If relevant, consider translating estimates of relative risk into absolute risk for a meaningful time period	N/A	

	Item No.	Recommendation	Page No.	Relevant text from manuscript
Other analyses	17	Report other analyses done – eg analyses of subgroups and interactions, and sensitivity analyses	N/A	
**Discussion**				
Key results	18	Summarise key results with reference to study objectives	7-8	In this study
Limitations	19	Discuss limitations of the study, taking into account sources of potential bias or imprecision. Discuss both direction and magnitude of any potential bias	7	Limitations
Interpretation	20	Give a cautious overall interpretation of results considering objectives, limitations, multiplicity of analyses, results from similar studies, and other relevant evidence	9	In summary
Generalisability	21	Discuss the generalisability (external validity) of the study results	7-9	Single centre
**Other information**				
Funding	22	Give the source of funding and the role of the funders for the present study and, if applicable, for the original study on which the present article is based	9	Financial/nonfinancial disclosure
